# Regulatory Role of Cell Division Rules on Tissue Growth Heterogeneity

**DOI:** 10.3389/fpls.2012.00174

**Published:** 2012-08-09

**Authors:** Karen Alim, Olivier Hamant, Arezki Boudaoud

**Affiliations:** ^1^School of Engineering and Applied Sciences, Harvard University Cambridge, MA, USA; ^2^Laboratoire de Reproduction et Développement des Plantes, Laboratoire Joliot Curie, Institut National de la Recherche Agronomique, CNRS, Ecole Normale Supérieure de Lyon, Université Claude Bernard Lyon 1 Lyon, France

**Keywords:** growth regulation, stochasticity, morphogenesis, mechanical forces, vertex model, cell division

## Abstract

The coordination of cell division and cell expansion are critical to normal development of tissues. In plants, cell wall mechanics and the there from arising cell shapes and mechanical stresses can regulate cell division and cell expansion and thereby tissue growth and morphology. Limited by experimental accessibility it remains unknown how cell division and expansion cooperatively affect tissue growth dynamics. Employing a cell-based two dimensional tissue simulation we investigate the regulatory role of a range of cell division rules on tissue growth dynamics and in particular on the spatial heterogeneity of growth. We find that random cell divisions only add noise to the growth and therefore increase growth heterogeneity, while cell divisions following the shortest new wall or along the direction of maximal mechanical stress reduce growth heterogeneity by actively enhancing the regulation of growth by mechanical stresses. Thus, we find that, beyond tissue geometry and topology, cell divisions affect the dynamics of growth, and that their signature is embedded in the statistics of tissue growth.

## 1 Introduction

The coordination of cell division and cell expansion lies at the heart of tissue growth in any organism. Plant cells are constrained by cellulosic walls and glued to their neighbors by a pectic lamella, therefore, the regulation of growth has to be achieved without cell migration or cell removal. This makes the orientation of cell divisions and the direction of cell expansion critical for normal development (Smith, 2001). While it is clear that the orientation of divisions changes the local geometry and mechanics of the tissue, its impact on tissue growth seems to have received little consideration. Here, we investigate whether division orientation contributes to growth regulation.

The patterns of cell division in plants have attracted attention for a long time. Initially observations of cell geometries ignited the idea that cell shape determines the cell division plane. It was noted that cells often form a new cell wall perpendicular to the axis of maximal cell expansion (Hofmeister, 1863), that the plane of division follows the shortest path dividing the cell into two equally sized daughter cell (Errera, 1888), and that new walls are nearly perpendicular to the existing ones (Sachs, 1878). Such models received renewed interest recently within the world of plants (Dupuy et al., 2010; Besson and Dumais, 2011) and beyond (Minc et al., 2011). On a different footing mechanical forces have been proposed to select the plane of division (Green, 1962, 1980; Hejnowicz, 1984) and can also be seen as a generalization of the cell geometry based rules (Hejnowicz, 1984). Mechanical forces have been observed to affect the direction of new cell walls in *in vitro* plant tissue cultures (Yeoman and Brown, 1971; Lintilhac and Vesecky, 1984; Lynch and Lintilhac, 1997) as well as the orientation of the mitotic spindle in *in vitro* animal cells (Théry et al., 2005, 2007; Fink et al., 2011). In particular, the alignment of cell division planes with the stress along the creases between the shoot apex and emerging organs, at the tip of plant shoots, suggests cell division to be parallel to the direction of maximal stress (Hamant et al., 2008).

All models for plant cell division are inherently linked to plant cell growth being dominantly a mechanical response of the encasing cell wall to the high osmotic turgor pressure within each cell (Szymanski and Cosgrove, 2009; Mirabet et al., 2011). It is the anisotropy in mechanical properties of the cell wall that initially gives rise to asymmetric cell expansion. Then the microtubules radiating from the nucleus can potentially measure cell shape and thus generate a geometric cell division rule (Flanders et al., 1990; Lloyd, 1991; Besson and Dumais, 2011). In addition the microtubule binding protein CLASP1 that localizes at highly curved cell walls could provide a readout of cell shape (Ambrose et al., 2011). On the other hand the turgor driven yielding of the meshwork of cellulose fibers within the cell walls creates a mechanical strain. These mechanical cues might be sensed and interpreted by the highly dynamic and regulated microtubule cytoskeleton and thus give rise to stress oriented cell division (Mirabet et al., 2011). Both cell shape and stress driven cell division plane location could in principle be present at the same time, with cell shape being more dominant at low stress levels and competing with stress driven realignment of microtubules at high stress levels for example. Eventually division planes are established through the assembly of microtubules and actin filaments in a cortical preprophase band that circumscribes the future division plane site (Mineyuki, 1999; Smith, 2001; Müller, 2012; Rasmussen et al., 2011).

**Figure 1:**
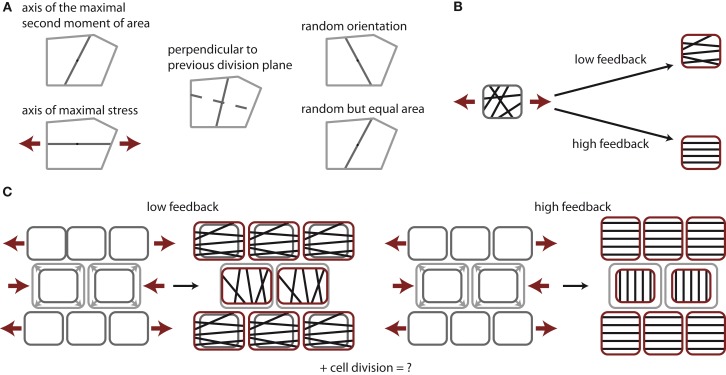
**Tissue growth control by cell division and stress feedback**. **(A)** Cell division rules under consideration. Cells form a new cell wall through the cell’s center of mass either along the axis of the maximum principal second moment or area, the axis of maximal stress, perpendicular to the previous cell division plane of the same cell, along a random axis, or along a random axis ensuring equal division in area. Note an increase in randomness in the cell division plane from left to right. **(B)** Impact of stress feedback on microtubule ordering within a cell. Mechanical stress experienced within a cell, illustrated by red arrows, aligns microtubules in parallel at high feedback, whereas only to some extent at low feedback. **(C)** Impact on stress feedback on growth homogeneity. At low feedback the stress arising due to the overgrowth of the center cells induces a stress field that slightly suppresses vertical growth in the center cells, while surrounding cells grow horizontally to maintain tissue contiguity, thus driving homogeneous growth. At high feedback cells cannot grow along the main direction of stress at all. The growth of the cells in the center is completely halted but surrounding cells continue to grow vertically, establishing steep gradients in growth between cells.

Microtubules coordinate not only the orientation of cell division but also the direction of cell expansion (Pastuglia and Bouchez, 2007; Sedbrook and Kaloriti, 2008; Lloyd, 2011). Cortical microtubules generally control the direction of cellulose deposition (Lloyd and Chan, 2008) and thus microtubule orientation prescribes the direction in which the cell wall is reinforced by cellulose fibers. The predominant orientation of the cortical microtubules depends on mechanical force fields (Green and King, 1966; Williamson, 1990; Cleary and Hardham, 1993; Zandomeni and Schopfer, 1994; Wymer et al., 1996; Ikushima and Shimmen, 2005; Elsner, 2008) and specifically aligns with the direction of maximal stress (Williamson, 1990; Hamant et al., 2008). Hence, cell walls resisting maximal (tensile) stresses are reinforced in a mechanical feedback mediated by the microtubule dynamics, see Figure 1 for an illustration. Such a mechanical feedback has been hypothesized for animal tissues as a mechanism to regulate the growth heterogeneity on a tissue scale (Shraiman, 2005; Aegerter-Wilmsen et al., 2007, 2010; Hufnagel et al., 2007). The shoot apical meristem of plants exhibits inherent growth heterogeneity (Kwiatkowska and Dumais, 2003; Grandjean et al., 2004; Kwiatkowska, 2004; Reddy et al., 2004) that has been attributed to differential elastic properties of cells (Milani et al., 2011; Peaucelle et al., 2011; Kierzkowski et al., 2012). Recent work shows that indeed the mechanical feedback potentiated by the dynamics of the microtubules is impacting this growth variability and thus morphogenesis (Uyttewaal et al., 2012).

Now the orientation of cell divisions poses a parallel lever of growth coordination in plant tissues. Comparisons of tissue growth models with tissue statistics show that the mode of cell division inherently affects the statistics of the geometry of a tissue (Patel et al., 2009; Aegerter-Wilmsen et al., 2010; Sahlin and Jönsson, 2010; Gibson et al., 2011) and thus potentially growth. Interestingly, cell division plane orientation can uncouple growth and patterning in plants. For instance, in the *tonneau* mutants, which do not exhibit a preprophase band, and consequently show random cell division plane orientation, cell identity is preserved, floral organs, and histological features being well positioned and recognizable (Traas et al., 1995). However, organ size is dramatically reduced in the *tonneau* mutants, and growth is largely isotropic, consistently with the lack of coordination in microtubule and cell division plane orientations.

Here we investigate whether the dynamics of growth could be coordinated by the orientation of cell divisions themselves. We therefore pursue the question of how cell divisions contribute to growth variability within a tissue simulation. We focus on isotropically growing tissue as present in the shoot apex (Kwiatkowska and Dumais, 2003; Reddy et al., 2004), as this meristematic tissue not only provides the precursor cells for aerial organs but also coordinates organ emergence. Note that a tissue growing overall isotropically does not imply that all individual cells are growing isotropically. Measurements indeed do indicate anisotropic growth in the shoot apex (Kwiatkowska and Dumais, 2003; Uyttewaal et al., 2012), which might guide the orientation of cell division planes. In modeling the dynamics of growth, we incorporate the reduction of growth in the direction of maximal stress as indirectly mediated by cortical microtubules. The impact of various cell division models on growth variability is tested. We confine ourselves to symmetric cell divisions as they appear to be the default scenario which is modulated by intrinsic and external stimuli. We assess the impact of cell divisions on tissue geometry and topology and on the dynamics of growth, as provided by the distribution of mechanical stresses and growth variability. We find that randomly oriented cell division planes merely enhance the noise during growth dynamics, while new walls dividing a cell along a shortest midplane or along the direction of maximal stress enhance growth regulation and reduce local tissue growth heterogeneity.

The details of the tissue growth simulation, the considered rules of cell division and their implementation are described in section 2. In the results section 3 we outline our findings on the impact of cell divisions on global tissue mechanics and local stress variability, which are discussed in section 4.

## 2 Materials and Methods

We investigate the effect of cell divisions on growth variability within a tissue growth simulation. The simulation aims to account for the contributions in growth coordination provided by both the control of cell expansion and the role of cell divisions. For the dynamics of cell expansion we assume that cell growth in the direction of maximal mechanical stress is reduced as implied by the cortical microtubule mediated feedback on cell walls. For the role of cell divisions we are less specific and compare a broad range of models for orienting a cell division plane.

As growth control via cell expansion and cell division is provided via cell geometry and tissue mechanics, we choose a level of modeling that allows to track individual cell shape and local mechanical stress with the least amount of free parameters. This is provided by a vertex model simulation, in which the state of the system is defined by all vertices only, while walls are assumed to be straight. As many epithelium-like tissues form a mono-layered tissue where cells only divide perpendicular to the surface, the growth dynamics of the epidermal layer of the shoot apical meristem is essentially two dimensional, allowing us to constrain the model to two dimensions. We neglect the effect of curvature because the model is aimed to describe local heterogeneity (between neighboring cells) in the central zone of the shoot apical meristem, where mechanical stress is predicted to be isotropic. If desired the impact of curvature can be incorporated in the simulation by adding the appropriate global stress field, assuming that relative values of cell turgor driven stress and organ shape driven global stress can be estimated.

In the following we detail the ingredients of the model.

### 2.1 Tissue growth

**Figure 2:**
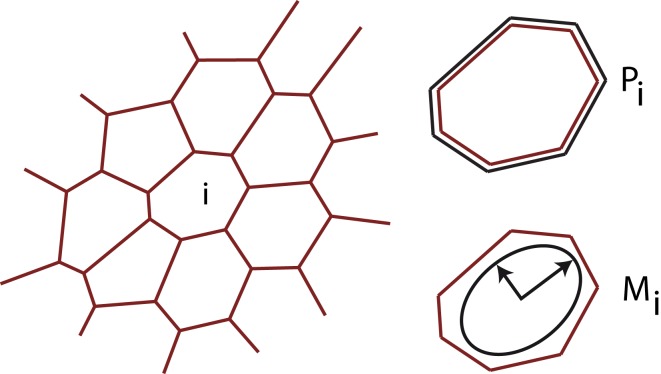
**Illustration of the tissue model**. The tissue is abstracted to a two dimensional tilling of space by cells, which are defined by straight cell walls intersecting in vertices, where three neighboring cells meet. At each time-point the tissue is in mechanical equilibrium, balancing the forces arising from differential growth. Mechanical forces are captured by an energy functional encompassing the perimeter *P_i_* of each individual cell *i* and an anisotropic form tensor *M_i_*, defined as the second moment of area tensor, allowing us to implement anisotropic individual cell expansion.

To model tissue growth we employ a generalized vertex model simulation as introduced in Uyttewaal et al. (2012). A tissue is represented as a flat polygonal tilling of space, where edges represent cell walls and vertices three cell contact points as sketched in Figure 2. The growth of each cell (numbered by *i*) is driven by the growth of an individual, anisotropic target ellipse represented by a matrix $M_{i}^{\left(0\right)}.$ The actual shape of a given cell yields a form matrix *M_i_* = (*M_i,*xx*_*, *M_i,*xy*_*, *M_i,*xy*_*, *M_i,*yy*_*) defined as the second moment of area of its *v* vertices with position *x*, *y*.

$$\begin{array}{ll} M_{i,xx}=\overset{v-1}{\underset{k=1}{\sum }}\frac{a_{k}}{12}\left(y_{k}^{2}+y_{k}y_{k+1}+y_{k+1}^{2}\right), & \\ M_{i,yy}=\overset{v-1}{\underset{k=1}{\sum }}\frac{a_{k}}{12}\left(x_{k}^{2}+x_{k}x_{k+1}+x_{k+1}^{2}\right), & \text{(1)}\\ M_{i,xy}=\overset{v-1}{\underset{k=1}{\sum }}\frac{a_{k}}{24}\left(x_{k}y_{k+1}+2x_{k}y_{k}+2x_{k+1}y_{k+1}+x_{k+1}y_{k}\right), & \end{array}$$

with *a_k_* = *x_k_y_k+1_* − *x_k+1_y_k_*. Positions and shapes of cells are determined from the condition of mechanical equilibrium balancing the quest of individual cells to attain their target ellipses. This is achieved by minimizing the total energy of a tissue encompassing *i* ∈ [1, *N*] cells.

(2) $$E=\overset{N}{\underset{i=1}{\sum }}\left\{\alpha P_{i}+\beta {\left(\text{Tr}\left[M_{i}-M_{i}^{\left(0\right)}\right]\right)}^{2}+\chi {\left(\text{Det}\left[M_{i}-M_{i}^{\left(0\right)}\right]\right)}^{2}\right\}.\;$$

By minimizing the difference in trace and determinant of actual cell form *M_i_* and target ellipse $M_{i}^{\left(0\right)}$ we ensure that the eigenvalues of the actual cells form are the closest possible to those of the target ellipse. The additional term that is proportional to the cell perimeter *P_i_* represents the tension in cell walls. The minimization of the total energy with respect to cell vertex positions determines not only cell position and shape within the tissue but also the local stress tensor *S_i_* exerted by surrounding cells on cell *i*. The definition of stress on a cell *i* follows from (Landau and Lifshitz, 1981) by calculating the force at every vertex *k* as the energy’s gradient *F_k_* = ▽*_k_E*, and interpolating the force at two vertices linearly along the edge between them,

$$\begin{array}{l} S_{i,xx}=\overset{v}{\underset{k=1}{\sum }}\left(\frac{F_{x,k}{\hat{x}}_{k}+F_{x,k+1}{\hat{x}}_{k+1}}{3A_{i}}+\frac{F_{x,k}{\hat{x}}_{k+1}+F_{x,k+1}{\hat{x}}_{k}}{6A_{i}}\right),\\ S_{i,yy}=\overset{v}{\underset{k=1}{\sum }}\left(\frac{F_{y,k}{ŷ}_{k}+F_{y,k+1}{ŷ}_{k+1}}{3A_{i}}+\frac{F_{y,k}{ŷ}_{k+1}+F_{y,k+1}{ŷ}_{k}}{6A_{i}}\right),\\ S_{i,xy}=\overset{v}{\underset{k=1}{\sum }}\left(\frac{F_{x,k}{ŷ}_{k}+F_{y,k}{\hat{x}}_{k}+F_{x,k+1}{ŷ}_{k+1}+F_{y,k+1}{\hat{x}}_{k+1}}{6A_{i}}\right.\\ \;\left.+\frac{F_{x,k}{ŷ}_{k+1}+F_{y,k}{\hat{x}}_{k+1}+F_{x,k+1}{ŷ}_{k}+F_{y,k+1}{\hat{x}}_{k}}{12A_{i}}\right),\text{(3)} \end{array}$$

where $\hat{x}$ and ŷ denote the relative vertex coordinates with respect to the cell’s center of mass, and *A_i_* expresses the cell’s area.

Growth is described by the time-evolution of $M_{i}^{\left(0\right)},$ and has two contributions. First, the basal growth rate γ(1 ± σ) exhibits stochastic fluctuations of amplitude σ (Uyttewaal et al., 2012). Second, based on the observation that cortical microtubules orient according to the highest stress, reducing growth in that direction, see Figures 1B,C, we model the effect of such a feedback on tissue growth by coupling a cell’s asymmetric stress component, the deviatoric stress *D_i_*, (defined by *D_i_* = *S_i_* − 1/2Tr[*S_i_*]∈ 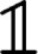, 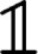
being the identity matrix). Therefore, the target ellipse changes in time as

(4) $$\frac{dM_{i}^{\left(0\right)}}{dt}=\gamma \left(1\pm \sigma \right)M_{i}^{\left(0\right)}-\frac{\eta }{2}\left(D_{i}M_{i}^{\left(0\right)}+M_{i}^{\left(0\right)}D_{i}\right).$$

η stands for the strength of the stress feedback and quantifies the capacity of cortical microtubules to reorient according to stress, and the speed at which this re-orientation occurs. As extension to incorporate anisotropic growth one can also interpret the basal growth rate as a tensor instead of a scalar and thereby enforce for example preferred growth along one direction of space.

### 2.2 Cell division

To study the regulatory role of different cell division modes we restrict ourselves to the case of roughly symmetric cell divisions by considering only cell divisions planes pivoted at the cell’s center of mass. Hence, cell division modes only differ in the cell division plane’s direction within a cell. The five cell division modes are sketched in Figure 1A, namely,

**Table:** 

**moment of area:**	along the axis of the maximum principal second moment of area, a representation of Errera’s rule (Errera, 1888), as the axis of the maximum principal second moment of area will point along the shortest axis of the cell,
**stress:**	along the axis of maximal stress,
**previous plane:**	perpendicular to the mother cell’s previous division plane,
**random:**	along a random direction,
**equal area:**	along a random direction that ensures exact splitting into to equal areas.

Cells divide once they have reached a threshold area *A*^(0)^. Upon cell division along axis $\vec{e}$ the target matrix $M_{m}^{\left(0\right)}$ of the mother cell is inherited by the daughter cells $M_{d_{i}}^{\left(0\right)},$ *i* = 1, 2, according to the following rules, which ensure that, in the coordinate system spanned by the division axis $\vec{e}$ and its orthonormal partner $\vec{n},$ the parallel component of the target matrix of each daughter cell equals the mother’s and the perpendicular component of the mother cell is split according to the ratio of the area of the daughter $A_{d_{i}}$ and the mother cell $A_{m},\;{\vec{e}}^{T}M_{d_{i}}^{\left(0\right)}\vec{e}={\vec{e}}^{T}M_{m}^{\left(0\right)}\vec{e},\;{\vec{n}}^{T}M_{d_{i}}^{\left(0\right)}\vec{n}=\frac{A_{d_{i}}}{A_{m}}{\vec{n}}^{T}M_{m}^{\left(0\right)}\vec{n}.$

### 2.3 Implementation

The simulation is implemented in a custom written C++ program. Simulation parameters are chosen as α = 0.02, β = 7.0, χ = 1.0, γ = 0.01, η ∈ [0, 15], and σ ∈ [0, 1], specifically values of σ = 0.5 and σ = 0.85 have been used for low and high intrinsic noise levels. Simulation results are independent of the precise choice of the tissue energy model parameters α, β, and χ as long as the relative order of magnitude between the line tension parameter α and the “elastic” parameters β, χ is kept at 10^−2^, as this ensures convexly shaped cells. Choosing the parameter γ amounts to setting the simulation time step and must therefore be chosen small enough to allow equilibration of the tissue after each growth step. In each simulation run the tissue is initialized as an isotropic hexagonal tissue consisting of three cells with their $M_{i}^{\left(0\right)}$ a factor of 0.7 smaller than their actual second moment of area matrix. The tissue is grown to a size between 150 and 200 cells undergoing as many cell divisions and between 20 and 60 cycles of growth for low and high values of feedback strength, respectively.

### 2.4 Observables

A main tissue characteristic is its local growth variability,

(5) $${\text{Gvar}}_{i}=\sqrt{\frac{\text{Tr}\left[\left(G_{i}-{\left\langle G_{i}\right\rangle }_{nn_{i}}\right){\left(G_{i}-{\left\langle G_{i}\right\rangle }_{nn_{i}}\right)}^{\text{T}}\right]}{\text{Tr}\left[{\left\langle G_{i}\right\rangle }_{nn_{i}}{\left\langle G_{i}\right\rangle }_{nn_{i}}^{\text{T}}\right]},}$$

which denotes the normalized variability in growth, measured by the growth tensor *G*, of an individual cell *i* relative to the mean of its own growth rate and its nearest neighbor’s growth rate ${\left\langle G_{i}\right\rangle }_{nn_{i}}.$ Taking the trace Tr of the tensors ensures a scalar observable. The growth tensor *G* itself is defined as the difference of the actual form matrix after a growth and subsequent energy minimization step normalized by the actual cell form,

(6) $$G_{i}\left(t\right)=\frac{M_{i}\left(t\right)-M_{i}\left(t-dt\right)}{M_{i}\left(t\right)}.$$

As further tissue characteristics we consider the local stress variability, which is defined according to the same formula as the growth variability evaluating a cell’s stress tensor *S_i_* instead. Furthermore, the stress anisotropy is calculated as the absolute difference between the largest and smallest eigenvalue of the stress tensor normalized by their sum. The tissue’s geometric and topological properties are assessed with the cell area, the number of edges per cell, a shape factor defined as area over perimeter squared, and as a measure of the positive opening angle between edges the squared opening angle at a vertex. Further, we measure the amount of stress released due to a cell division and subsequent energy minimization as,

(7) $$\Delta S=\frac{1}{N}\overset{N}{\underset{i=1}{\sum }}\left\{\frac{\sum_{<i>}\text{Tr}\left({\tilde{\text{S}}}_{i}{\tilde{\text{S}}}_{i}^{T}\right)\;-\sum_{<i>}\text{Tr}\left(S_{i}S_{i}^{T}\right)\;}{\sum_{<i>}\text{Tr}\left({\tilde{\text{S}}}_{i}{\tilde{\text{S}}}_{i}^{T}\right)\;+\sum_{<i>}\text{Tr}\left(S_{i}S_{i}^{T}\right)\;}\right\},$$

where <*i*> denotes the sum over cell *i* and its nearest neighbors and $\tilde{S}$ and *S* stand for the stress before and after cell division, respectively.

## 3 Results

**Figure 3:**
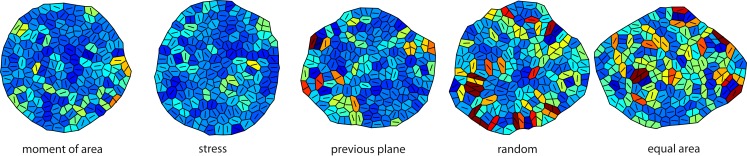
**Snapshots of the tissue simulation for different cell division modes**. The color code corresponds to increasing growth rates going from dark blue to dark red. The line within each cell marks the direction of maximal growth. Strikingly the spatial variability of growth and the anisotropy of the overall tissue shape increases from left to right accompanied by individual cell shape being less circular. Data shown are tissues comprising about 150 cells grown at a feedback of 14.

We started by investigating the impact of the five cell division modes (moment of area, stress, previous plane, random, and equal area, as defined in Figure 1) on cell mechanics. Because cell contiguity is preserved the different cell division modes impact very differently on tissue mechanics and therefore give rise to alternative tissues growth dynamics and resulting tissue geometry and topology. In the following, we present and analyze our simulations, which are illustrated in Figure 3.

### 3.1 Local stress release by cell division

**Figure 4:**
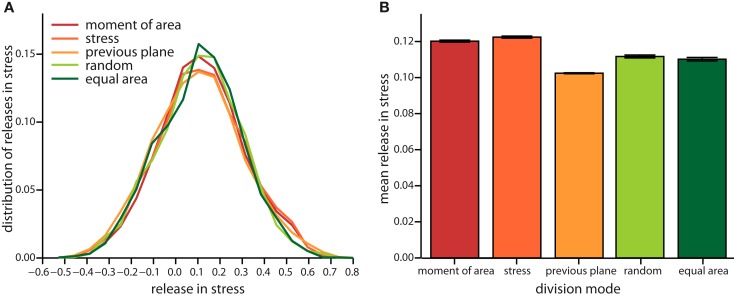
**Local release in stress due to the different cell division modes**. **(A)** Distribution of the total change in stress in the dividing cell and its nearest neighbors. Negative (respectively positive) stress means that stress increases (respectively decreases) following division. Subtle differences in the distribution are revealed in the mean local release of stress shown in **(B)**. Release is largest for cell division along axis of maximum principal stress and maximum principal second moment of area. Equally but less stress is released for random and equal area cell division modes. Cell divisions perpendicular to the previous plane of cell divisions release least stress. Data shown comprises at least 35,000 individual cells for each division mode. The qualitative trend is almost independent of feedback, see Figure A3 in the Appendix, data displayed is for a feedback of 14.

On the very local level the direction of a cell division plane instantaneously affects the stress distribution within the tissue. In the dividing cell itself the new cell wall increases stress following division, as the new wall like any cell wall in the tissue bears tension. But this cell wall also affects the mismatch between target and actual cell form of the dividing cell and, hence, opens room for neighbors to reduce their mismatch as well. Thus including the neighborhood, stress is most often released on average in the group of cells comprising the dividing cell and its nearest neighbors, as shown in Figure 4. The mean amount of released stress is always positive but the total amount is different between the cell division modes. Noteworthy, a cell division plane oriented along the direction of maximum principal stress releases most stress, shortly followed by a plane following the shortest new wall (implemented by the axis of the maximum principal second moment of area). Less stress is released by random cell division or cell divisions that are random but ensure exact equal splitting of areas. The least stress is released for cell divisions along the direction perpendicular to the previous division plane. Therefore, the role of cell divisions appears to be to release stress; however the precise amount of release can be regulated by the type of division mode. From the observations of stress variability discussed in the next section it also seems plausible that cell division modes such as “second moment” and “stress” that respond to cell mechanics can specifically release stress in high stress regions. As the stress is coupled via the dynamics of the cortical microtubules to the growth dynamics, the magnitude, and localization of the release of stress should therefore be reflected in the growth dynamics.

### 3.2 Global tissue mechanics

**Figure A1:**
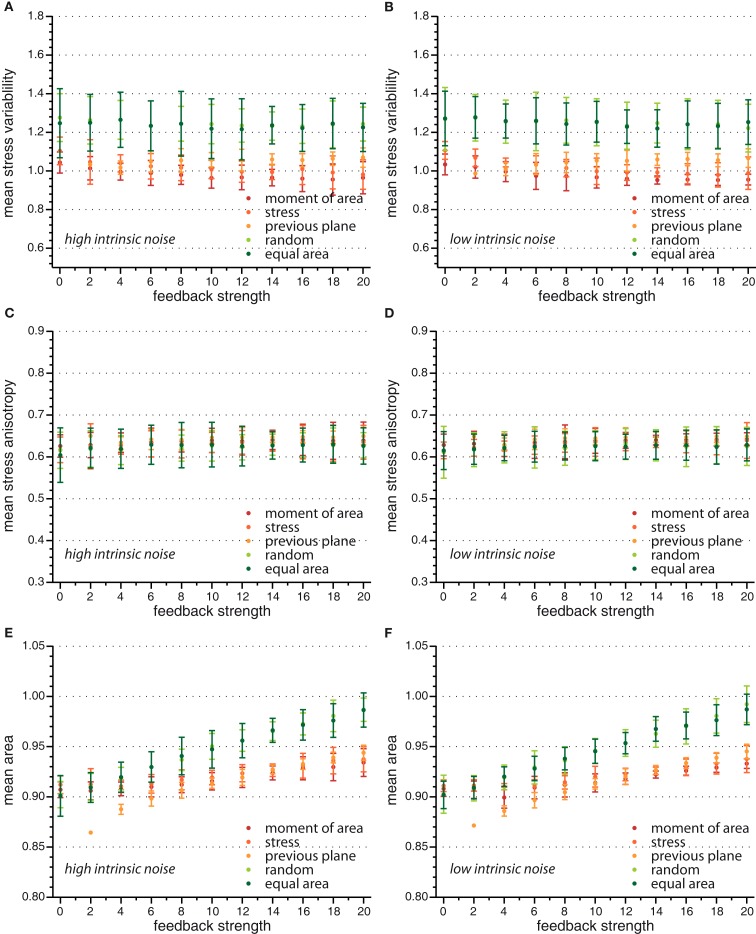
**Mean value of observables characterizing tissue geometry, topology, and stress distribution for the different cell division modes versus feedback strength for high and low intrinsic noise**. While mean stress variability **(A,B)** and mean stress anisotropy **(C,D)** do not display a significant dependence on feedback strength, the mean area **(E,F)** is increasing linearly with feedback strength.

**Figure A2:**
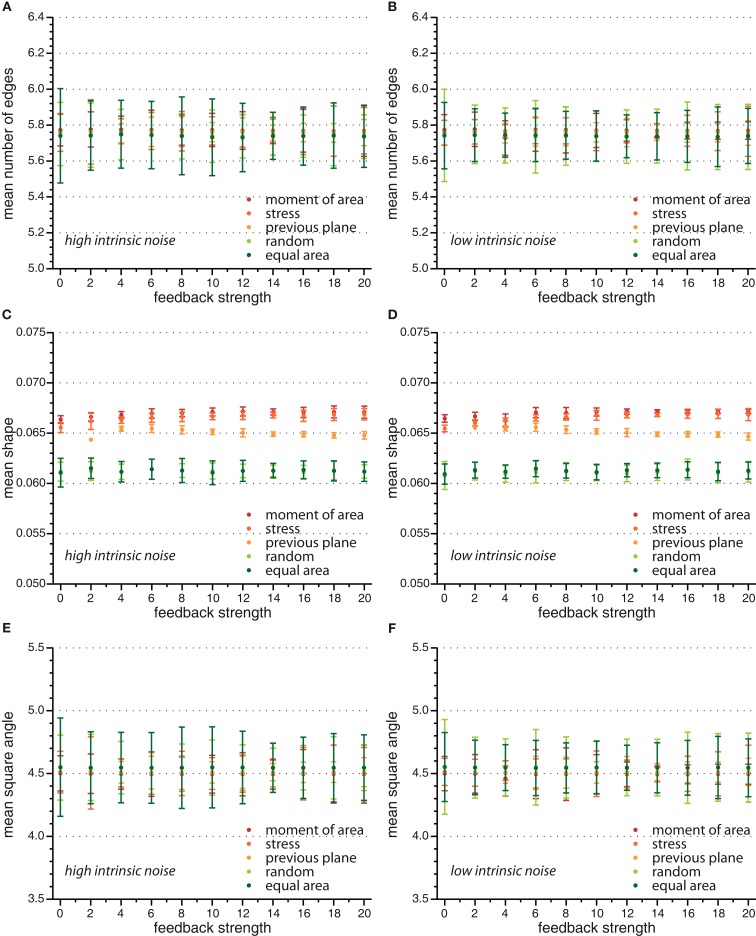
**Mean value of observables characterizing tissue geometry, topology, and stress distribution for the different cell division modes versus feedback strength for high and low intrinsic noise**. Here mean number of edges **(A,B)**, mean shape **(C,D)**, and mean square angle **(E,F)** are independent of feedback strength.

On the global scale of the whole tissue the differences in cell division modes also affect tissue mechanics, and overall geometry and topology. Table 1 display (i) mechanical properties such as the mean stress variability and the mean stress anisotropy, and (ii) tissue geometry and topology such as mean area, mean number of edges, mean shape, and the quadratic mean of the opening angle. The average displayed is taken over the whole range of stress feedback values considered. Apart from small variations of the mean stress anisotropy and the stress variability only the mean area shows a significant linear increase with feedback strength, see Figures A1 and A2 in the Appendix. As cells divide at a threshold area of one, the increase of the mean area with feedback strength implies that the distribution of cell sizes becomes narrower at larger feedback, i.e., faster growth. Concerning mechanical variables, we found no variations in the mean stress anisotropy as a function of cell division mode while the stress variability is about 20% lower for “moment of area,” “stress,” and “previous plane” compared to “random” and “equal area” division mode. The reason for this difference can only partly be attributed to stress release, as when cells divide perpendicular to a previous division plane least stress is released but the stress variability lies below the one of “random” or “equal area” cell divisions. However, stress variability correlates very well with growth heterogeneity, which is discussed in the following section. Finally, concerning the tissue’s geometrical and topological parameters we find no variations in the mean squared opening angle and the mean number of edges as expected on pure topological grounds. The mean area and the mean shape on the other hand show that divisions guided by the second moment of area, stress, or previous division plane lead to smaller and more compact, circular shaped cells, as observed in Sahlin and Jönsson (2010) and exemplified in the snapshots in Figure 3. Notably the level of intrinsic noise does not affect these findings, see Figures A1 and A2 in the Appendix.

**Table 1:** **Mean value of observables characterizing tissue geometry, topology, and stress distribution for the different cell division modes at high and low intrinsic noise level**.

Low intrinsic noise	Division mode				
Observable	Moment of area	Stress	Previous plane	Random	Equal area
Stress variability	0.99 ± 0.06	1.02 ± 0.06	1.06 ± 0.04	1.24 ± 0.1	1.24 ± 0.1
Stress anisotropy	0.64 ± 0.03	0.63 ± 0.03	0.65 ± 0.02	0.62 ± 0.03	0.63 ± 0.05
Area	0.915 ± 0.009	0.920 ± 0.008	0.929 ± 0.005	0.953 ± 0.01	0.950 ± 0.02
Number of edges	5.77 ± 0.09	5.77 ± 0.09	5.76 ± 0.05	5.74 ± 0.1	5.74 ± 0.2
Shape	0.0669 ± 0.0004	0.0665 ± 0.0004	0.0649 ± 0.0003	0.0612 ± 0.0007	0.0613 ± 0.0009
Squared angle	4.5 ± 0.2	4.5 ± 0.1	4.5 ± 0.1	4.5 ± 0.2	4.5 ± 0.3

**High intrinsic noise**	**Division mode**				

Stress variability	0.96 ± 0.03	1.03 ± 0.04	1.06 ± 0.04	1.25 ± 0.1	1.25 ± 0.1
Stress anisotropy	0.64 ± 0.02	0.63 ± 0.02	0.65 ± 0.02	0.62 ± 0.04	0.62 ± 0.03
Area	0.921 ± 0.006	0.917 ± 0.01	0.930 ± 0.005	0.946 ± 0.01	0.945 ± 0.01
Number of edges	5.77 ± 0.06	5.77 ± 0.06	5.76 ± 0.05	5.74 ± 0.2	5.74 ± 0.1
Shape	0.0670 ± 0.0002	0.0665 ± 0.0002	0.0649 ± 0.0003	0.0611 ± 0.0009	0.0612 ± 0.0007
Squared angle	4.5 ± 0.1	4.5 ± 0.1	4.5 ± 0.1	4.5 ± 0.2	4.5 ± 0.2

*Average is taken over all feedback strengths considered. Notably stress variability is about 20% lower for cell divisions along the maximum principal second moment of area or stress or for cell divisions perpendicular to the previous cell division plane than in any other case. Also the area of cells in these two cases is smaller and the shape more circular. There are no significant changes between the cases of high and low intrinsic noise level*.

### 3.3 Growth heterogeneity

**Figure 5:**
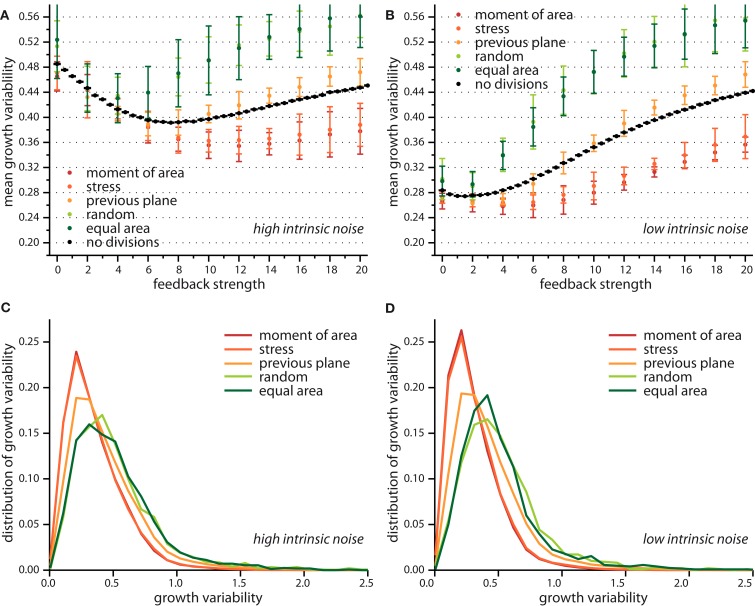
**Growth variability versus feedback strength for different cell division modes**. Mean growth variability for high **(A)** and low **(B)** intrinsic noise and the respective distributions of growth variability at a feedback of 14 for each case, in **(C,D)** respectively. As reference the pure impact of feedback strength (without any cell division) is shown in black. Cell divisions along the maximum principal second moment of area or stress enhance the effect of a growth feedback reducing the growth variability below the value observed for no cell divisions. Cell divisions with high noise on the other hand, where cells divide along a random direction, or randomly but ensuring equal areas counteract the effect of stress feedback yielding higher growth variability. The case where the cell division plane is perpendicular to the previous divisions plane interpolated between those two limiting cases. Distribution of growth variability is significantly broader at high noise, however differences between cell division modes are subtle.

Taken together the release of stress on the local scale and the tissue wide characteristics already suggest different impacts on growth dynamics for the two modes of division according to moment of area or to stress, compared to the two random modes of division. The case of a division plane oriented perpendicular to the previous division plane seems to be a chimera, releasing least stress on the one hand but giving rise to tissue properties that resemble those for the two other deterministic division modes, which release most stress. This chimeric behavior is indeed reflected qualitatively in the snapshots of Figure 3 and quantitatively in the change of the mean growth variability with feedback strength displayed in Figure 5. In the latter Figure, we compare the mean growth variability of the different division modes with a tissue grown without cell divisions, hence, affected only by intrinsic noise and intrinsic feedback. At high intrinsic noise level the deviatoric stress driven feedback decreases growth variability significantly before it increases again at high feedback. This amplification of differences in growth due to high feedback is even more striking for low intrinsic noise level, where the local minimum of most homogeneous growth is shifted to very small feedback strength. Cell divisions now quantitatively (but not qualitatively) modify the response of the growth variability to feedback strength. Cell divisions with a random direction or a random direction selected to split a cell into equal areas decreases the impact of stress feedback, increasing growth variability. Cell divisions along the maximum principal axis of the second moment of area or stress on the other hand enforce the impact of feedback strength, yielding a more homogeneous growth throughout. Cell division planes perpendicular to the previous division plane approximately follow the mean growth variability of a tissue without cell divisions. Variability in growth is also nicely reflected in the overall tissue shape as presented from simulation snapshots in Figure 3. Hence, we find that cell division modes do have a regulatory role in tissue growth dynamics. As the change in growth variability between different division modes reaches 30%, it is interesting to speculate whether a spatial dominance of one division mode over the other could give rise to spatial patterns in local growth variability as observed in the shoot apex (Uyttewaal et al., 2012), where for instance growth appears more heterogeneous at boundaries.

## 4 Discussion

Plant tissues have two ways to regulate their growth, on the one hand by coordinating cell expansion and on the other hand by directing the orientation of cell division planes. Both pathways seem to be regulated by mechanical stresses within the tissue. Cell expansion is asymmetric, reduced in the direction of maximal stress due to a mechanical feedback on cell wall strengthening mediated by cortical microtubules. The orientation of cell division planes often agrees with the direction of the cell’s shortest midplane (Besson and Dumais, 2011) or the direction of maximal stress (Hamant et al., 2008). As mechanical stresses change and adapt during the growth of a tissue, these feedbacks from mechanical stresses on the tissue mechanics itself provide an autonomous way to impact not only the final tissue morphology but also the tissue growth dynamics.

It has been shown that the mechanical feedback on cell expansion can provide a mean to reduce or amplify growth heterogeneities in tissues (Uyttewaal et al., 2012). Here, we addressed how the orientation of cell divisions impacts a tissue’s growth dynamics. We considered two noisy rules of cell division namely random orientation of cell division planes and random orientations that divide the cell in exactly two halves. Further, we assessed three deterministic cell division rules: division perpendicular to the previous cell division plane, along the maximum principal second moment of area (roughly corresponding to the shortest new wall) and along the direction of maximum principal stress. We find that random cell divisions release less stress within the tissue, create larger, and more asymmetric cells with higher stress variability and growth variability than those cell divisions following the direction of maximum principal second moment of area or stress. In contrast, when cell shape or stress prescribe the cell division plane, cell shape is more isotropic and stress variability is the least among all rules – possibly indicating the sensitivity of growth regulation in those cases. The comparison of the growth variabilities with the reference cases of no cell divisions shows that the random rules increase the variability above reference, while new walls aligned with the direction of maximum principal second moment of area or stress regulate growth and decrease the variability below reference. The case of the deterministic rule where cells divide perpendicular to the previous plane of cell division more or less follows the reference growth variability confirming no clear participation in either of the two other groups as also observed in global tissue statistics.

**Figure 6:**
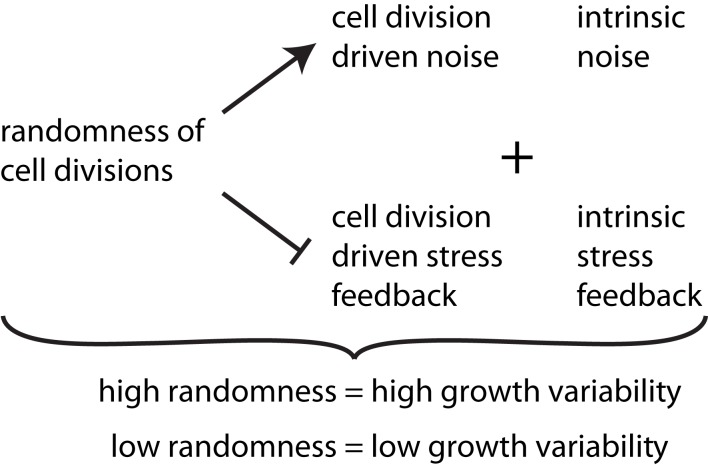
**Synopsis of the effect of cell divisions on growth variability**. The degree of randomness of cell divisions induces noise in addition to intrinsic noise in individual growth rates of cells. Thereby it reduces the effect of stress feedback arising from both cell division driven and intrinsic stress feedback. High randomness of cell division thus leads to high tissue growth variability while tissue reinforcement in the direction of maximal stress, and thus contributes to stress feedback in addition to intrinsic feedback, which decreases growth variability.

From our observations the level of noise within a cell division rule arises to be the determining factor of how much regulation this rule can provide, see summary in Figure 6. There seems, however, to be a counter example in the case of the “previous plane” division rule, that is indeed very deterministic by definition but does not provide additional growth regulation. In fact, cell division planes that appear always perpendicular to the previous division plane are not affected by the growth dynamics of the tissue itself. Therefore, this division rule inherently carries the same noise as the stochasticity of cell growth itself and cannot provide any regulatory role. Only cell division rules which interact with the growth of a tissue, either by reacting to cell expansion or mechanical stresses can regulate tissue growth dynamics. In those cases a tissue autonomous regulation of growth is provided. These different rules might reflect a way for cells to modulate their growth. In particular, specific cellular mechanisms can be associated with cell division rules. For instance, it has been observed that plant cells often loose their preprophase band when grown as cell cultures. Consequently, spindle and phragmoplast orientations are more variable in those cells (Chan et al., 2005). This suggests that the preprohase band may act as a sensor of the behavior of neighboring cells, and thus the presence of a preprophase band in a cell might reflect a certain level of interaction with the growth of the tissue. It is also tempting to speculate that the choice of cell division mode might be spatially regulated in the developing plant to achieve regions of more irregular growth contrasting regions of very regulated, smooth growth. Indeed growth variability varies with the distance from the tip at the shoot apical meristem (Uyttewaal et al., 2012), suggesting a role for growth regulation in organ emergence.

While the case of random cell divisions might appear a bit extreme in the context of development, the choice of the cell division mode might nevertheless be regulated, even when restricted to the rules that depend on an interaction with the tissue. Cell division along a cell’s shortest midplane, which we implemented according to the direction of maximum principal second moment of area, has been found to be in agreement with large scale tissue statistics (Sahlin and Jönsson, 2010; Besson and Dumais, 2011). However, there exist examples where this rule performs badly, namely in the crease between the shoot apical meristem and an emerging organ cell divisions occur according to the longest axis of the cell. There the orientation of new cell walls agrees with the direction of maximum principal stress (Hamant et al., 2008). The analysis presented in this work shows that both cell divisions along the shortest midplane and along the axis of maximal stress give rise to equivalent tissue geometry, topology, and tissue growth dynamics. We have, however, restricted ourselves to the case of isotropically growing tissue as present only at the tip of the shoot apical meristem. So our conclusion is that isotropic growth cannot provide the data to discern between both rules. A distinction is in our opinion only possible in a tissue that grows anisotropically. Growth data of the shoot apical meristem would, however, be suitable to substantiate the general principle arising from our work, namely that the randomness in the orientation of cell division regulates tissue growth variability. In practice one could investigate mutants in which the orientation of the new wall is abnormal, such as *ton1* (Traas et al., 1995) or *pok1;pok2* (Müller et al., 2006; see also Müller, 2012 for a review) and compare their shoot apex tissue statistics and most importantly growth dynamics in a similar setup as in Uyttewaal et al. (2012). Experimentally challenging but a very interesting continuation would be to study shoot apices that are for example mechanically perturbed or spatially constrained so that they grow anisotropically. In such a setting the distinction between divisions guided by maximal stress or the shortest midplane would be possible. The nature of the perturbation or constraint can rigorously be added to the simulation presented here as outlined in section 2. Also specific differences in cell’s elastic properties can be in implemented in the simulation to investigate the role of spatial differences in cell mechanics as found in Milani et al. (2011), Peaucelle et al. (2011), Kierzkowski et al. (2012).

In summary we show that the orientation of cell division does not only contribute to tissue geometry and topology but also to the dynamics of tissue growth. Cell division planes that are oriented along the cell’s shortest midplane or the direction of stress actively contribute to the tissue growth dynamics by releasing stress and thus reducing stress variability and growth variability within a tissue. While geometric and topological tissue characteristics are limited to discern between different modes of cell division, measuring quantities such as growth variability provides handles to distinguish cell division modes and beyond learn about the regulation of growth via the orientation of cell division.

## Conflict of Interest Statement

The authors declare that the research was conducted in the absence of any commercial or financial relationships that could be construed as a potential conflict of interest.

## Figures and Tables

**Figure A3:**
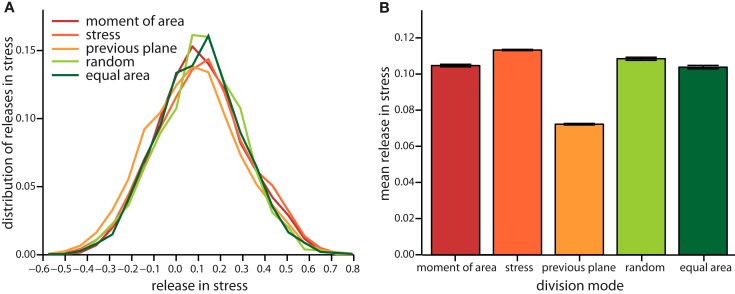
**Local release of stress due to the different cell division modes as in Figure 4 but for a feedback of 4**. **(A)** Distribution of the total change in stress in the dividing cell and its nearest neighbors and the respective mean versus division mode **(B)**.
